# Tumor-Associated Regulatory T Cells in Non-Small-Cell Lung Cancer: Current Advances and Future Perspectives

**DOI:** 10.1155/2022/4355386

**Published:** 2022-04-22

**Authors:** Jiaqi Liang, Guoshu Bi, Guangyao Shan, Xing Jin, Yunyi Bian, Qun Wang

**Affiliations:** Department of Thoracic Surgery, Zhongshan Hospital, Fudan University, Shanghai 200032, China

## Abstract

Non-small-cell lung cancer (NSCLC) is one of the most threatening malignant tumors to human health, with the overall 5-year survival rate being less than 30%. Regulatory T cells (Tregs), a functional subset of T cells, maintain immunologic immunological self-tolerance and homeostasis. Accumulating evidence has uncovered their implicated roles in various cancers in recent years. In NSCLC, they are associated with staging, therapeutic efficacy, and prognosis by infiltrating in tissues and thereby attenuating immunologic anticancer effects in patients. Tumor-associated Tregs display distinct immune signatures in NSCLC compared to thymus-derived Tregs, playing an important role in remodeling the tumor microenvironment (TME). Targeting Tregs has become a novel direction for NSCLC patients, such as disrupting their immune-suppressive functions, blocking their trafficking into tumors, and inhibiting their development and/or activation. This review is aimed at elucidating the molecular mechanisms of tumor-associated Tregs in NSCLC and providing therapeutic targets relevant to Tregs.

## 1. Epidemiology and Prevention of NSCLC

Lung cancer is one of the most commonly diagnosed cancers and the leading cause of cancer death worldwide in the year 2020, with an estimated 2.2 million new cases and 1.8 million deaths, which represents more than one in ten (11.4%) cancers diagnosed and approximately one in five (18.0%) deaths [1]. Traditionally, 85% of all cases are histopathologically classified as non-small-cell lung cancer (NSCLC) [2]. While treatment options comprise surgery, chemoradiotherapy, and targeted therapy, patients with NSCLC are often diagnosed with metastatic diseases or develop resistance to the drugs, resulting in a frustrating five-year overall survival of only less than 30% currently [3–5].

In recent years, tumor immunity has become a hot spot. The emergence of immunotherapy led by anti-immune checkpoint molecules has improved the overall survival of a subset of patients with advanced NSCLC. However, patients with positive tumor PD-L1 expression may still experience poor outcomes and severe adverse effects or even have a deterioration of their disease defined as hyperprogression [6, 7]. In recent years, accumulated evidence has uncovered the implicated roles of regulatory T cells (Tregs) in various cancers. In this review, we review the latest progress in the mechanisms of Tregs in promoting tumors and propose perspectives and therapeutic strategies targeting Tregs in NSCLC.

## 2. Summary of Suppressive Mechanisms of Tregs

Tregs, an immunosuppressive subset of CD4-positive (CD4+) T cells, are first found to serve to maintain immunological self-tolerance and homeostasis. Accumulated evidence has uncovered their implicated roles in autoimmune diseases and cancer [8–12]. They can be classified into two major groups based on their developmental origin, including thymus-derived Treg cells (tTregs) and peripheral Treg cells (pTregs) in vivo or induced Treg cells (iTregs) in vitro [12–14].

In the development of tTregs, several downstream signaling pathways are activated through the interaction between CD25 molecule (also known as interleukin-2 receptors, IL-2R*α*), which are highly expressed on the membrane [14–16], and interleukin-2 (IL-2) [17], resulting in an increase of *FOXP3* expression [18–20]. Elevation of *FOXP3* can further promote the level of immunosuppressive receptors (i.e., CTLA-4, TIGIT, LAG-3, NRP1, CD39, and CD73) expressed on Tregs and enhance the secretion of multiple inhibitory cytokines such as IL-10, TGF-*β*, and IL -35, thus endowing Tregs with an immunosuppressive function [21–23]. In contrast, under costimulation of IL-2, TGF-*β*, or other cytokines, peripheral CD4+ T cells can be differentiated into a new cell subset with highly expressed *FOXP3* that are designated pTregs or iTregs [24–27].

**Figure 1:**
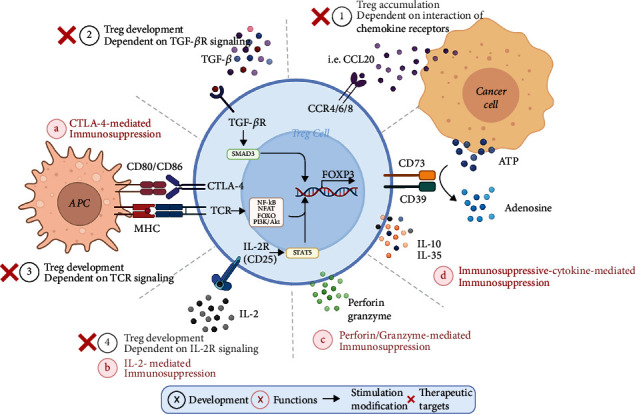
Prime mechanisms of Treg-mediated immunosuppression and associated therapeutic targets in NSCLC. Black: in the tumor microenvironment (TME), the development of regulatory T cells (Tregs) depends on several main factors that contribute to the *FOXP3* expression: (1) the interaction of chemokine receptors, (2) TGF-*β*R signaling between Treg and cancer cells, (3) TCR, and (4) IL-2R signaling in Tregs. Red: Tregs express the high-affinity IL-2 receptor binding to and sequestering IL-2 to reduce its availability to effector T cells. They also express cytotoxic T lymphocyte antigen 4 (CTLA-4), which binds to CD80 and CD86, with a higher affinity than CD28, on antigen-presenting cells (APCs), thereby transmitting suppressive signals to these cells. In addition, Tregs can produce immunosuppressive cytokines, granzymes, and perforin to inhibit immunity. Red cross: different therapeutic approaches have been explored in downregulating Treg cell expansion mediated by chemokine or TGF-*β* in the TME. In addition, inhibitors targeting TCR and IL-2R signaling have been tested in reducing Treg cell activation and proliferation in patients.

Effector Treg cells (eTregs), characterized by their high ability to suppress immunity, perform immunosuppressive functions mainly through cell contact-dependent and cytokine-mediated pathways. Cytotoxic T lymphocyte antigen 4 (CTLA-4) and IL-2R are two key molecules that mediate this process (Figure 1). eTregs can inhibit the costimulatory signals in effector T cells by expressing membrane and producing soluble CTLA-4, which can bind to CD80 or CD86 molecule that is expressed on antigen-presenting cells (APCs) [28–32]. Besides, eTregs can hamper the activation and tumor-killing capacity of CD8+ T cells and natural killer (NK) cells via depriving IL-2 owing to its high-affinity receptor CD25 on the cell surface [33–35]. They can also attenuate the function of APCs and effector T cells by secreted or intracellular inhibitory molecules, such as IL-10, TGF-*β*, and IL-35, or cell-killing factors such as granzyme and perforin (Figure 1) [36–39].

## 3. Current Advances of Tumor-Associated Tregs in NSCLC

### 3.1. Infiltration of Tumor-Associated Tregs and Tumor Prognosis

Tregs are associated with oncogenesis, invasion, metastasis, reoccurrence, drug responses, and prognosis of patients in multiple cancers by remodeling the immune-suppressive microenvironment [40]. Tumor-associated Tregs account for 10–50% of CD4+ T cells in tumors, with only 2–5% of those in the peripheral blood of healthy individuals by contrast [12, 41]. However, they have displayed distinct immune signatures and activated immunophenotypes. Three groups of tumor-associated Tregs have been found in tumors, including tumor-resident, tissue-resident Tregs, and those from the circulation [42–44]. However, their origins and relationship are still unclear that whether tumor-infiltrated Tregs originate in the tumor-associate tissues or from the circulation. Treg infiltration has been a negative prognostic factor for patients with NSCLC. Increased number and enhanced activity have been found with Tregs in multiple tissues of patients, including tumors, metastatic lymph nodes, and the peripheral blood, which is highly associated with the staging and the occurrence of metastasis and recurrence NSCLC [45–47].

Besides, the frequency of Tregs in NSCLC contributes to resistance or even hyperprogressive diseases after chemotherapies and immunotherapies. Liu et al. [48] reported that the efficacy of platinum-based chemotherapy in NSCLC decreased with the increasing ratio of *FOXP3*+ Treg and CD8+ T, suggesting the abundance of eTregs in tumor sites was an independent factor for poor response to platinum-based chemotherapy. Consistent results were also found in a mouse model. Pircher et al. [49] demonstrated that the number of *foxp3*+ Tregs could increase when treated with platinum-based chemotherapy combined with cetuximab. eTregs potently attenuated the NK-mediated anticancer effects and the antibody-dependent cell-mediated cytotoxicity (ADCC) against CD8+ T cells.

### 3.2. Characteristics of Tumor-Associated Tregs and Tumor Prognosis

Compared to the natural tTregs, the key features of tumor-associated Tregs are highly activated and differentiated effector Tregs, with higher affinity to the T cell receptor (TCR). Transcriptomic data showed that several immune checkpoints (i.e., interleukin-1 receptor 2, PD-1 ligands, and CCR8 chemokine) were upregulated to maintain their suppressive role in tumors [50]. Higher amounts of immunosuppressive molecules can increase and expand in tumor tissues, inducing stronger immune suppression [41, 43, 44, 51].

It is worth noting that members of the TNF receptor superfamily (TNFRSF) also play a crucial role in the development and maturation of Tregs, which has not been detailed in our previous text. Molecules such as GITR (also known as TNFRSF18), OX40 (also known as TNFRSF4), and TNFR2 (also known as TNFRSF1B) can function as costimulatory agents in regulating the expression of the FOXP3 gene [52]. In NSCLC, TNFR2+ Tregs presented in the peripheral blood and pleural effusion of NSCLC patients were more proliferative and expressed a higher degree of CTLA-4 molecules to mediate immunosuppression than TNFR2- Tregs [53, 54]. Furthermore, TNFRSF9 was proposed to promote the immune-suppressive activity of Tregs in the TME in NSCLC [55]. We still need to identify new molecules of Tregs in order to better isolate them and explore their roles in the TME.

### 3.3. Tumor-Associated Tregs in the TME

Different cells and molecules in the TME also provide favorable conditions for tumor-associated Tregs to exert immunosuppressive functions (Figure 1). Recent studies in several kinds of tumors revealed that insufficient glucose supply and increased intracellular glycolysis in cancer cells could provide Tregs rich in lactic acid and fatty acids, promoting their proliferation [56, 57]. Besides, TGF-*β*, ATP, IDO, and some other molecules produced by tumor cells also enhanced the immunosuppressive function of Tregs in tumor tissue [58–60]. In NSCLC, tumor cells from patients with higher disease stage or lymph node metastasis generated more TGF-*β* than their counterparts [61], which could not only expand the infiltration of eTregs in the TME but potentiate the immunosuppressive function by elevating the expression of inhibitory molecules B7H1 and GITRL on the surface of APC cells [62].

Apart from the above metabolites, tumor cells can also escape from immune-mediated tumor surveillance by recruiting Tregs by expressing a variety of chemokines (Figure 1). Zhang et al. [63] reported that CCL20 secretion by tumor cells could recruit Tregs via cooperating with its receptor CCR6 in NSCLC. Moreover, the TME can posttranslationally regulate the expression of *FOXP3* in Tregs. It has been shown that the AREG protein secreted by tumor cells of lung adenocarcinoma can maintain the Treg suppressive function via the EGFR/GSK-3*β*/FOXP3 axis in vitro and in vivo [64].

Several studies have shown that oncogene mutations can regulate the differentiation process of Tregs. One of the most studied has been *KRAS*, a fundamental driver of lung tumorigenesis, generally affecting 20-40% of NSCLC patients. The incidence is higher in smokers than in nonsmokers (30% vs. 10%) [65]. *KRAS* mutations in lung cancer cells were found to promote the differentiation of more CD4+ T cells into Tregs in the TME by increasing the secretion of TGF-*β* and IL-10, thereby increasing the number of cells in the TME [66, 67].

The progression and metastatic capacity of solid tumors are also influenced by some other immune cells in the TME. Macrophage receptor with collagenous structure (MARCO) expressed on the surface of tumor-associated macrophages (TAM) has been reported to promote the proliferation of Tregs and the inhibitory cytokine IL-10 secretion in NSCLC [68]. Besides, at the protumor inflammatory stage of lung cancer, TGF-*α* stimulation can upregulate MHC-II molecules on the surface of alveolar type II cells to trigger Treg expansion and promote the tumorigenesis of inflammation-driven lung adenocarcinoma [69].

## 4. Research Progress and Clinical Applications of Targeting Tumor-Associated Tregs

Because Tregs play roles in tumor immunity, measurements targeting them have emerged in tumor treatment in recent years, and three categories of approaches have been explored: disrupting their immune-suppressive functions, blocking their trafficking into tumors, and inhibiting their development and/or activation. Currently, therapeutic strategies targeting Tregs in oncology including NSCLC often involve two or more of the above to enhance antitumor immunity.

### 4.1. Disrupting Treg Cell Immune-Suppressive Functions in NSCLC

Monoclonal antibodies against cell membrane markers of Tregs have been currently the most commonly used method to inhibit the IL-2-mediated immunosuppressive function of Tregs (Figure 1). Given the constitutive and high expression of CD25 by most Tregs and its crucial role in eTreg cell maintenance, CD25 has attracted attention as a potential target in Treg depletion [70–73]. In clinical studies, CD25-blocking monoclonal antibody daclizumab administration has led to a marked and prolonged decrease in Tregs in patients with melanoma [74]. In NSCLC, preclinical results demonstrated that Treg depletion blocked by CD25 in combination with cytotoxic therapy might be beneficial as a treatment strategy. Ganesan et al. [75] found that mice bearing early NSCLC treated with daclizumab and chemotherapy exhibited significantly increased tumor cell death and extended survival associated with infiltration CD8+ T cells.

### 4.2. Blocking Treg Trafficking into Tumors in NSCLC

Previous studies have revealed mechanisms that lead to intratumoral Treg accumulation involve the interaction of chemokine receptor-expressing activated Tregs and the chemokines produced in the TME (Figure 1) [12, 76–78]. CCR4, a key chemokine receptor highly expressed on the surface of Tregs, has been a promising target in oncology herein [43, 79]. A monoclonal antibody, mogamulizumab, which depletes CCR4+ Tregs, could suppress tumor growth, having been approved for adult T cell leukemia or lymphoma [79].

However, the antitumor efficacy of this drug in solid tumors is unclear due to the less amount of surface CCR4 expression on Tregs in the TME. Indeed, clinical trial results did not show a significant synergistic antitumor effect for combined therapy using mogamulizumab and docetaxel or nivolumab, an anti-PD-1 agent, in advanced and perioperative NSCLC patients (https://www.clinicaltrials.gov/, NCT trial numbers: NCT02358473, NCT02946671), which may be attributed to the small number of patients enrolled. Recently, efficacy and safety of Treg depletion with mogamulizumab in combination with immune checkpoint inhibitors (anti-CTLA-4 or PD-1 or PD-L1 molecules) are being explored and determined in numerous kinds of advanced solid tumors, such as liver cancer, gastric cancer, and pancreatic cancer (NCT trial numbers: NCT02281409, NCT01929486, and NCT02476123), on which we could pin our hopes. In addition, CCR4 is not only expressed on Treg but similarly on the surface of conventional T cells, bringing about off-target immune side effects. These data highlight the need to identify molecules specific to tumor-accumulated Tregs as therapeutic targets.

Promisingly, there have still been several alternative molecules associated with Treg cell recruitment that may also effectively prevent tumor progression in solid tumors. The study conducted by Alvisi et al. [80] reported that transcription factor IRF4 could bind to BATF, reducing Treg cell recruitment by regulating the expression of chemokine receptors on Tregs. Besides, IRF4 can also downregulate the expression of inhibitory factors in Tregs, such as TNF receptor superfamily molecules and ICOS, thereby inhibiting the immunosuppression in the TME. Generally, chemokines are produced by cells in the TME, including tumor cells and TAMs, which can also be combated to reduce Tregs' accumulation. Researchers have found that docetaxel could rescue immunity functions against tumor cells by reducing their secretion of CCL20 interacted with CCR6+ Tregs [63].

### 4.3. Inhibiting Treg Development and/or Activation in NSCLC

Several directions have been considered to deactivate and convert Tregs into effective T cells enhancing immunity to various cancers. These include nonspecific cytotoxic agents, strategies selectively targeting molecules important for Treg differentiation and maturation and regulating gene expression in the nucleus of premature T cells.

#### 4.3.1. Nonspecific Cytotoxic Agents

Traditional chemotherapeutics, such as cyclophosphamide, are effective in Treg depletion. Cyclophosphamide could significantly reduce the number of circulating Tregs in the peripheral blood of colorectal cancer patients [81, 82]. Low-dose cyclophosphamide combined with CD25 monoclonal antibody had better efficacy than the anti-CD25 mouse model with NSCLC receiving radiotherapy [83]. Furthermore, whether the addition of cytotoxic agent, cyclophosphamide or doxorubicin, can improve the efficacy of anti-PD-1 therapy by modulating tumor environment in NSCLC patients with PD-L1 expression less than 10% remains unclear to be figured out (NCT trial number: NCT03808480).

#### 4.3.2. Treg Signaling Pathway Inhibitors

Two tyrosine kinase inhibitors, imatinib and dasatinib, have been found to inhibit LCK molecules on the surface of T cells as an off-target effect, impairing maintenance and immunosuppressive activity of Tregs by blocking TCR signaling (Figure 1) [84]. Redin and his colleagues reported that dasatinib could synergize with PD-1 inhibitor to impair tumor growth in NSCLC experimental mouse models. They uncovered that inactivated SFK targeted by dasatinib could inhibit the phosphorylation of STAT5 and SMAD3, which were downstream molecules of CD25 and TGF-*β*R, respectively, hereby inhibiting the TGF-*β*-induced differentiation process of Tregs. However, the combination of dasatinib and EGFR-TKI drug did not prolong the survival time of patients with EGFR mutations in NSCLC [85–87]. Its benefit remains to be determined when combined with PD-1 antibody in patients with advanced NSCLC (NCT trial number: NCT04284202, NCT02750514).

The phosphoinositide 3-kinase pathway (PI3K) is an important aspect of Treg cell development and function, which mediates signaling downstream of the TCR [88–90]. The inhibitor's deficiency of PI3K*δ* by inhibitor could inhibit Treg cell activation and augment immunity to control cancer in mice via CD8+ T cells [91, 92]. Ahmad et al. reported a consistent result in a mouse lung cancer model that anticancer vaccine coadministered with the PI3K*δ* inhibitor reduced the number of T cells whereas the number of effector T cells increased, leading to a decrease in tumor volume [91]. The efficacy and safety results of PI3K*δ* inhibitor INCB050465 combined with pembrolizumab are currently being evaluated in a phase I trial in patients with advanced tumors, including NSCLC (NCT trial number: NCT02646748).

#### 4.3.3. Molecules Mediating Treg Cell Development

The transformation between the immunosuppressive Tregs and immune cells has been a hotspot in tumor immunity fields. Notably, there is a close relation between Treg and Th17 cells, where the Th17 cell subset has been discovered with similar properties to Tregs, both originated from a common precursor. Recent studies uncovered the association between their balance and the progression in different kinds of tumors [93–95]. Th17/Treg ratio was lower in patients' tumors and peripheral blood tissues than in healthy individuals, as demonstrated in studies focused on various solid tumors, including NSCLC [96–98].

Interestingly, cytokines in the TME are important in the differentiation of CD4+ T cell subsets, which depend upon the balance of expression of certain transcriptional factors. For instance, TGF-*β* can inhibit the differentiation of Th17 cells while inducing more Treg precursor cells to differentiate into Tregs via elevating *FOXP3* expression. On the contrary, the mediation of cytokines such as IL-1𝛽 and IL-6 contributes to Tregs secreting more increased amounts of IFN-*γ* and IL-17 and losing their original immunosuppressive function. This finally transforms Tregs into another two subsets of cells, Th1 or Th17, which will mediate immune clearance and inflammatory responses, respectively [99–101].

Mechanistically, Yu et al. [102] discovered that interferon regulatory factor 4 (IRF4) could induce more Th17 cells than Tregs in the malignant pleural effusion of patients with NSCLC via downregulating the expression of *HELIOS*, one of the dominant genes in Treg cell development. Moreover, curcumin has also been found to promote the conversion of Tregs to Th1 cells in NSCLC by inhibiting the transcription of *FOXP3* and promoting the expression of IFN-*γ*, which is necessary for Th1 cells [103]. Hence, inducing the differentiation of Tregs into other T cell subsets harboring antitumor functions can be promising in addressing tumors within the TME to be explored in clinical practice.

## 5. Future Perspectives

Treg infiltration into tumors is a contributor to poor prognosis via hindering effective immune response against tumor cells in NSCLC. Recent research has uncovered molecular mechanisms underlying their antitumor immunity. Despite the monumental advance of checkpoint blockade immunotherapy, high morbidity and mortality and different responses in NSCLC patients necessitate the consideration of alternative targets, where Tregs are most notably modulating immunosuppressive cells in the TME. Therefore, we should explore therapeutic targets against Tregs to reinvigorate cancer immunity in cancer treatment.
